# Sustainability assessment of waste vacuum systems as a collection solution

**DOI:** 10.1177/0734242X251385787

**Published:** 2025-11-13

**Authors:** Eirill Bø, Kari-Anne Lyng

**Affiliations:** 1 BI Norwegian Business School, Oslo, Norway; 2 NORSUS Norwegian Institute for Sustainability Research, Kråkerøy, Norway

**Keywords:** Urban waste management, vacuum waste system, underground containers, indoor bins, sustainability assessment, life cycle assessment, economies of scale

## Abstract

Efficient urban waste collection requires solutions that balance environmental performance, economic feasibility and social acceptability while minimizing spatial and ecological impacts. This study assesses the sustainability of vacuum waste collection systems in comparison with underground containers and indoor bins, using data from approximately 2500 households in a Norwegian city. Four waste fractions: plastic, residual and paper/cardboard were analysed across two scenarios: a fully vacuum-based system for all households, and a hybrid solution combining underground containers for 1500 households with indoor bins for 1000. The evaluation integrates three sustainability dimensions: climate impact through life cycle assessment, economic performance through lifecycle cost analysis and social aspects through qualitative assessment. Findings reveal that vacuum systems achieve the lowest climate impact, although the differences between systems are modest. For vacuum systems, emissions are dominated by the construction of pipe networks and terminal facilities; for underground containers, by the number of units and for indoor bins, by transport and operational activities. Economically, vacuum systems incur 22% higher costs than the hybrid solution. However, economies of scale are evident: increasing connected households from 2500 to 3100 reduces costs by 14%, positioning vacuum systems more competitively. Socially, vacuum systems offer notable benefits, including reduced odour and noise, improved traffic safety and decreased physical strain on personnel. Overall, the study concludes that waste collection solutions must be tailored to site-specific conditions but highlights the scalability and social advantages of vacuum systems, which may render them a viable option for sustainable urban waste management when integrated with commercial streams.

## Introduction

Municipal solid waste (MSW) is important from a sustainable perspective, it affects people, the planet and the economy in the collection system (Pires et al., 2019). Collection and transportation are regarded as crucial components of MSW management systems. They play a key role in terms of cost, public health impacts due to emissions, material recovery or recycling and resource depletion ( Heidari et al., 2019; Rodrigues et al., 2016; Yadav and Karmakar, 2020). Norwegian municipalities are tasked with the responsibility of collecting household waste to ensure the recirculation of valuable resources and to prevent waste from generating emissions or causing harm to natural ecosystems. Despite the environmental significance, the process of waste collection itself imposes certain burdens on the environment and residential areas, including emissions, noise and increased traffic. These collection activities furthermore represent a financial cost met by households through waste disposal fees. The total costs associated with collection and transportation are crucial and account for a significant share of the overall costs related to household waste management (Ghose et al., 2006; Yadav et al., 2016).

Recent advances in applied life cycle assessment (LCA) studies have provided critical insights into optimizing municipal waste collection systems. Notably, Bala et al. (2021) demonstrated the value of integrating environmental and operational data to benchmark collection strategies, revealing substantial opportunities for reducing emissions and improving resource efficiency. Incorporating such contemporary LCA findings strengthens the analytical foundation of this study and ensures alignment with current best practices in sustainable MSW management (Bala et al., 2021).

In the context of single-family homes, waste is typically collected in bins positioned outside the residence. However, urban environments necessitate more comprehensive solutions that can serve a larger number of homes while remaining unobtrusive and conserving valuable space within residential settings. The most pertinent waste collection solutions for such urban areas are indoor bins, underground containers and vacuum systems. Vacuum system minimizes the reliance on vehicle transportation in collection areas, helping to reduce noise, traffic congestion and potentially freeing up space (Kogler, 2007).

Indoor bins involve placing waste containers in shared waste rooms within buildings. These containers are then moved to a designated area for collection by waste trucks, which transport the waste for further processing. Underground containers consist of a chute above ground that leads to an inner container situated underground. These are emptied by crane trucks for subsequent waste processing. Vacuum systems involve a similar chute mechanism above ground, but these connect to a subterranean pipe system through which waste is transported using air suction to a terminal building. In this facility, waste is segregated into different fractions housed in separate containers, which are then collected by hook trucks for further treatment.

The principal aim of this article is to enhance knowledge that assists in making informed decisions regarding the selection of waste collection solutions in urban locales. This objective is operationalized through a comprehensive evaluation of the sustainability of vacuum systems relative to the collection methods. The sustainability assessment is multifaceted and encompasses three critical dimensions: potential climate impact, ascertained through LCA; cost analyses, which cover the full lifecycle and adhere to LCA phases and a simplified appraisal of social aspects, derived from literature reviews and insights from key stakeholders.

The analyses concentrate on the management of four distinct types of waste: food waste, plastic waste, residual waste and paper/cardboard. Two analyses form the core of the study. The first is a comparative assessment of the three collection solutions, namely indoor bins, underground containers and vacuum systems. The second analysis focuses on two area scenarios within a specific residential zone in Oslo, known as Bjørvika, comprising around 2500 homes. This area bifurcates into Bispevika, housing about 1000 homes currently employing indoor bins and Grønlikaia, with approximately 1500 homes slated for underground solutions. Here, a full-area vacuum system (scenario 1) is compared against a combination of indoor bins and underground containers (scenario 2).

The analysis relies on data sourced from Envac, the vacuum system provider, and Oslo municipality, specifically Oslo REG, which oversees the collection and treatment of household waste in Oslo. The comprehensive analysis aims to foster a deeper understanding of the relative sustainability performance of each system, guiding future choices in urban waste management solutions.

## Methodology

The analysed scenarios and the methods used to assess the three sustainability aspects (climate, economic and social impacts) are described in the following sections.

### Description of scenarios

The analyses in this article are based on the Bjørvika residential area, located in central Oslo, as described in section ‘Introduction’. This location is relevant as a study object because it is an urban area currently under development, where several of the collection solutions have already been implemented and thus allowing for collection of real data. The estimated annual waste volumes generated by the households in the area was calculated using the ‘Guidelines for Placement and Selection of Waste Management Solutions’ (Table 1; Renovasjons og gjenvinningsetaten, 2024).

**Table 1.** The amount of waste of different scenarios.

Waste type	Per housing unit (L week^−1^)	2500 housing units (tonnes year^−1^)
Residual waste	80	1144
Plastic packaging	30	390
Food waste	10	384
Paper and cardboard	50	1073
Total		2990

The amounts of waste are assumed to be the same across all collection systems.

The two scenarios for Bjørvika residential area are illustrated in Table 2. Currently, Oslo requires separate collection units for food waste when underground or vacuum systems are implemented. For vacuum systems, a separate collection unit for plastic is also required. Therefore, two alternatives for underground solutions (three and four separate collection units) and two alternatives for indoor bins (two and three separate units) were analysed.

**Table 2.** Description of the two scenarios.

Area	Waste fraction	Collection units	Operation	Transport	Infrastructure
Scenario 1					
Bispevika and Grønlia combined 2500 households	Residual, plastic, food, paper and cardboard	One chute for each fraction	Energy use for vacuum system	Hook truck on biogas	Establishment of terminal building and pipe system and excavation of pipe
Scenario 2					
Bispevika 1000 households	Residual, plastic and food	660-L plastic container	Placement of containers	Waste collection truck on biogas	Not affected
	Paper and cardboard	Same	Same	Same	Same
Grønlia 1500 households	Residual, plastic	Underground container	None	Crane truck on biogas	Excavation work related to installation of underground containers
	Food	Same	Same	Same	Same
	Paper and cardboard	Same	Same	Same	Same

Vacuum waste collection systems use underground pipes and air pressure to transport waste from collection points to a central terminal. These systems reduce the need for waste collection vehicles, thereby lowering traffic congestion and emissions. They also offer good hygiene, as the closed system prevents odours and pests, and they are space-efficient and aesthetically pleasing in dense urban environments. However, they require very high investment costs and complex maintenance. The systems are inflexible to urban development or demographic changes, and their operation is energy-intensive and vulnerable to technical malfunctions.

If a vacuum system was installed for all 2500 residents in Bispevika, the residents would deposit their waste into separate chutes for each of the four waste types, following Oslo Municipality’s guidelines (Energigjenvinningsetaten, 2018). The waste is transported via underground pipes to a terminal building. For the 2500 residents, it was assumed that 62 chutes were required. The chutes were assumed to have a lifespan of 20 years, a material consumption of 0.572 tonnes of steel/chute and an investment cost of 163,000 Norwegian kroner (NOK)/chute based on information from the technology provider (Lyng et al.).

The operation of the vacuum system requires energy for transporting waste between chutes and containers in the terminal building, as well as for ventilation and heating needs in the terminal. The energy consumption for the vacuum system is estimated at 299,000 kWhour year^−1^, based on an average of 100 kWhour tonne^−1^ of waste, with a total waste volume of 2990 tonnes year^−1^. Ventilation and heating needs amount to 60,893 kWhour year^−1^, based on an 825 m² terminal building. The electricity price is set at 1.5 NOK per kWhour.

Once the containers in the terminal building are full, a hook truck transports the waste to further processing (8.5 km). This truck is assumed to run on biogas. The biogas consumption is estimated at 0.37 kg km^−1^, with the truck having a lifespan of 10 years and a container capacity of 10 tonnes, based on information from the waste management department of municipality of Oslo (Lyng et al. 2022). Based on waste volume and truck capacity, the total number of trips required per year is 301.

Infrastructure includes groundwork, the terminal building (including containers) and pipes from chutes to the terminal. The investment cost for the terminal is 50.1 million NOK, with a lifespan of 60 years. The pipe system has a total length of 890 m and a lifespan of 60 years.

Installation of underground containers is possible for the 1500 residents under construction in Grønlikaia, but not for the 1000 existing residential units in Bjørvika due to space restrictions. The collection unit consists of a concrete and steel structure. The analyses were conducted for the separate collection of three and four types of waste. The number of chutes for four separate waste types is estimated at 120 units, serving 30–50 homes per chute based on maximum utilization. For three waste types, the required number of chutes is 90, following the same calculation method. Each concrete structure weighs 5.3 tonnes per chute, consisting of 96% concrete and 4% reinforcing steel, with a lifespan of 60 years. The steel container weighs 0.94 tonnes per chute, whereas the stainless-steel chute is 0.2 tonnes per chute, made of chromium steel, with steel components having a lifespan of 20 years. The installation cost per chute is 100,000 NOK.

Underground waste containers are installed below ground level, with visible above-ground inlets. They are emptied using crane-equipped vehicles. These containers offer large storage capacity, reducing the frequency of emptying. They maintain better hygiene due to the cooler underground temperatures, which limit odour and pest issues. Their design also improves the urban landscape by reducing visual clutter. On the downside, they require special vehicles for emptying and involve significant groundwork during installation. They also require adequate space and can be susceptible to freezing in colder climates.

The biogas consumption for the truck is 0.37 kgkm^−1^, with a lifespan of 7 years and a capacity of 7 tonnes. The round-trip travel distance is 8.5 km each way, with a local driving distance per trip of 4.1 km, assumed to be the same as for indoor bins. The number of trips is estimated at 257, based on waste volume and truck capacity.

Infrastructure for underground collection includes excavation work and the installation of underground containers. Excavation work per group is estimated at 120 m³ per group, with an excavation cost of 2500 NOK per m³, assuming a depth of 3 m. Infrastructure-related costs and environmental impacts for waste rooms were not included in the analysis, though using basement space for waste storage reduces available space for other uses, such as bike parking or storage.

Traditional waste bins are above-ground containers placed outdoors or in the basement, typically emptied manually. They are cost-effective and easy to install, with flexibility that allows them to be adapted to various areas and needs. Their low technical complexity makes them reliable and straightforward to manage. However, they can contribute to visual clutter and reduced urban aesthetics, have limited capacity requiring more frequent emptying and generally offer poorer hygiene with higher exposure to odours and pests.

Approximately 1000 homes in Bispevika currently rely on conventional waste bins placed in shared waste rooms. The input factors for indoor bin collection include 584 bins for two waste types, calculated based on Oslo Municipality’s recommendations, and 334 bins for three waste types, which include a separate bin for food waste. The bin size is 660 L for paper and residual waste, and 140 L for food waste. The materials used for the 660-L bins weigh 39 kg per bin and are made of high-density polyethylene, whereas the 140-L bins weigh 10.6 kg each and are made of the same material. The cost per 660-L bin is 2000 NOK, and the cost per 140-L bin is 625 NOK. The lifespan of the bins is estimated at 15 years.

Indoor bins require additional work to move bins from waste rooms to collection points. A forklift is used for this purpose. The building’s caretaker reported 20 hours week^−1^ to handle the bins and the forklift electricity consumption is 6.5 kWhour hour^−1^, information from Hangcha Electric Forklift.

The waste is collected by a collection truck running on biogas and transported to Haraldrud. The following assumptions are considered for the analysis: the biogas consumption of the truck is 0.37 kg km^−1^, with a lifespan of 7 years and a weight of 4.95 tonnes for a two-axle collection truck. The truck’s round-trip travel distance between Bispevika and Haraldrud is 8.5 km each way, totalling 17 km per trip. Additionally, the truck covers a local driving distance of 4.1 km per trip. Oslo’s current collection system involves separate collection of paper and cardboard, whereas food waste, plastic waste and residual waste is sorted into coloured bag (green, purple and white/black), which are collected in a shared collection unit and sorted at an optical sorting facility.

### Assessment of potential climate impacts

The climate impact of the collection systems was assessed using LCA. This is a recognized method for quantifying the environmental impact of products and services throughout their lifecycle. The steps in an LCA project are defined in ISO 14040 and 14044: defining goals and scope, inventory analysis (including data collection), environmental impact assessment and interpretation of results (14040, 2006; 14044, 2006). The goal and scope definition are crucial as it determines system boundaries, the type of data to collect, the indicators to assess and the context for interpreting the results. Results from an LCA are presented per functional unit, a quantified unit describing the system’s function. The functional unit ensures a fair comparison of the analysed systems.

The LCA evaluates the climate impact of vacuum systems compared to underground solutions and waste bins.

For the area scenarios in Bjørvika, the functional unit is defined as: ‘Collection of residual waste, paper/cardboard, plastic, and food waste generated by 2500 homes in Bjørvika over one year’.

**Figure 1.**
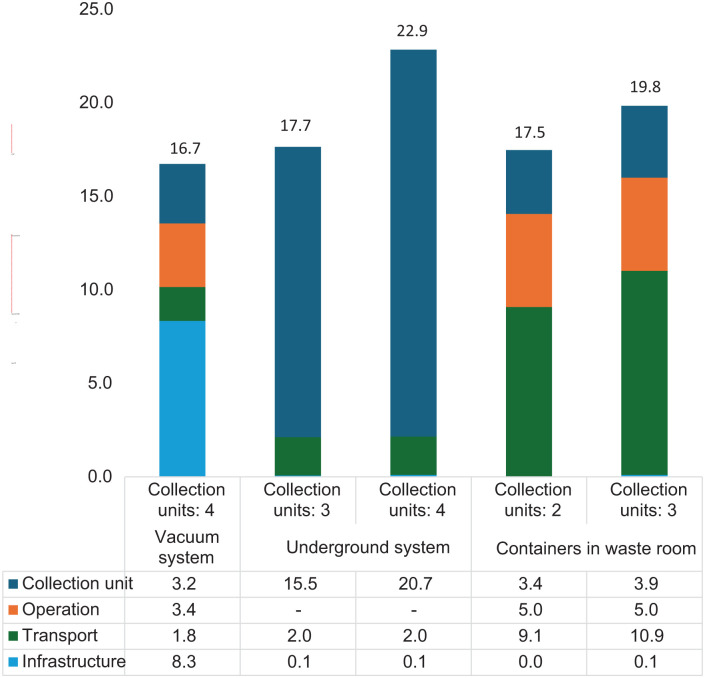
Kilogram of CO_2_-equivalents per household per year.

The system is divided into four lifecycle phases: collection units, operation of the collection system, transport and infrastructure, illustrated in Figure 1. The system was modelled in the LCA software SimaPro (Pré, 2024), and the database Ecoinvent cut off by classification v 3.10 was used for background data. The potential impacts from climate change are measured in kilograms of CO₂-equivalents using IPCC 2021 factors (100-year perspective). The LCA model developed in this project can also be used to examine other environmental indicators in the future. (Ref, supplemented materials)

### Assessment of costs

The costs are divided into four categories corresponding to the four lifecycle phases defined in the climate assessment: costs related to collection equipment, system operation, transport and infrastructure. Results are presented as annual costs. Investment costs are amortized over the expected lifespan.

Detailed transport calculations were performed for each solution. Waste collected using indoor bins, underground containers and vacuum systems employs different vehicles: collection trucks, crane trucks and hook trucks, respectively. Cost calculations for each vehicle type were based on data from Oslo REG and historical figures. Costs were divided into three components: fixed, variable and labour costs.

Fixed costs include depreciation, interest, insurance and administration, distributed over the annual kilometres driven per vehicle. Variable costs include fuel, maintenance and tyres. These are calculated per kilometre. Labour costs are based on estimated time usage, including driving time, collection time and unloading time.

### Qualitative assessment of social impacts

The evaluation of social aspects within the framework of social LCA (S-LCA), as outlined by the United Nations Environment Programme (UNEP, 2020), emphasizes the examination of both positive and negative impacts that a system may have on people. In this study, a simplified evaluation was conducted, adhering to the methodological steps prescribed by S-LCA. These steps include defining the assessment’s goals, identifying pertinent stakeholder groups, selecting indicator categories and evaluating the various impacts.

The primary objective of this social aspect analysis is to identify social advantages and disadvantages for the stakeholder groups for the analysed waste collection systems.

In terms of stakeholder identification, four primary groups were deemed relevant for the waste collection process: the local residential community, the users of the collection system, waste collection workers and municipalities or waste management companies. Each of these groups interacts with the waste collection systems in distinct ways and thus are impacted differently by the various solutions being assessed.

To engage with these stakeholder groups effectively, a simplified literature review was conducted. This review facilitated the selection of key indicator categories pertinent to each stakeholder group. The subsequent qualitative assessment of these categories drew from the literature and incorporated insights from key stakeholders actively engaged in or affected by the waste collection systems. This approach enabled a holistic evaluation of the social aspects associated with each waste collection solution, offering insights that can inform decisions in urban waste management practices.

## Results for the three collection solutions

The analysis compares the three collection solutions: vacuum systems, underground containers and indoor bins. Results are presented per household per year. The City of Oslo already requires the establishment of separate collection units for food waste when installing underground waste systems or vacuum waste collection. For vacuum systems, an additional separate collection unit for plastic is required. Due to these differences, we have analysed two alternatives for underground solutions: three and four separate collection units, and two alternatives for collection using containers in waste rooms: two and three separate collection units.

Figure 1 illustrates the potential climate impact of the three collection solutions. The results are expressed in kilograms of CO₂-equivalents per household per year.

The study’s results demonstrate that vacuum systems exhibit the lowest climate impact among the assessed waste collection solutions; however, the differences in climate impact among the systems remain relatively modest when viewed within the parameters established for this analysis. Each waste collection method has distinct sources contributing to its overall emissions profile. For vacuum systems, the most significant emissions originate from the construction and establishment of the pipe network and terminal building, with these emissions distributed over the entire lifespan of the system. In the case of underground containers, the number of collection units plays a pivotal role in determining emissions, as the production of these units is identified as the primary environmental burden. Although excavation work required for installation is accounted for under infrastructure, its influence is negligible in shaping the final emission results. For indoor bins, transportation emerges as the leading emission contributor, primarily due to the logistics involved in relocating waste. This is closely followed by emissions from system operations, particularly those related to the use of electric forklifts necessary for the movement of bins. These insights into emissions distribution provide a nuanced understanding of the environmental impacts associated with each waste collection approach.

**Figure 2.**
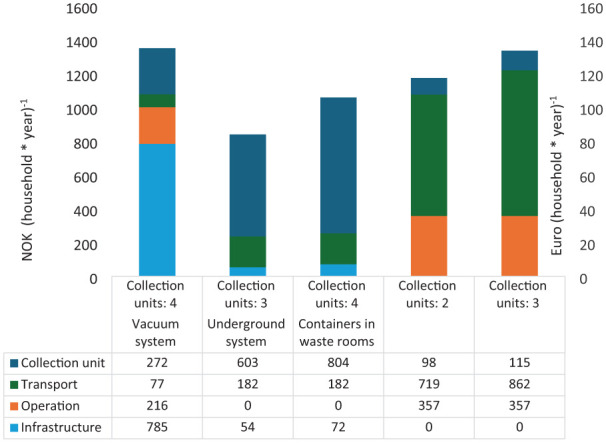
NOK and Euro (household × year)^−1^. NOK: Norwegian kroner.

Figure 2 illustrates the lifecycle costs of the three collection solutions, expressed in NOK and Euro per household per year.

The analysis of key cost drivers associated with each waste collection solution reveals distinct financial burdens and opportunities for optimization. In the case of vacuum systems, infrastructure investment represents the predominant cost factor, encompassing the construction of terminal buildings and the extensive pipe systems necessary for their operation. For underground containers, the primary financial burden stems from the high costs of the collection units themselves, reflecting their production and installation requirements. Conversely, for indoor bins, the costliest component is associated with the logistics of transportation facilitated by collection trucks, followed closely by the ongoing maintenance and operational activities related to the bins.

A sensitivity analysis highlights the economic potential inherent in vacuum systems, particularly their dependency on the number of connected households and the potential inclusion of commercial waste. Notably, the study found that increasing the number of connected homes from 2500 to 3100 results in a 14% reduction in costs per household, leading to a cost-efficiency that approaches the level observed for underground container systems with four units in operation.

Social impact in terms of social aspects, these were assessed qualitatively across various stakeholder groups to ascertain the broader societal impacts of each waste collection method. For the local residential community, vacuum systems offer significant social benefits, such as reduced odour and noise levels, as the transport and handling of waste occur outside residential areas. This advantage mitigates disturbances commonly associated with waste management. Underground containers similarly contribute to reduced odour issues because the waste is securely stored underground, making it less susceptible to the elements and minimizing exposure to residents. In contrast, indoor bins present potential challenges, including odour problems within waste rooms and during the collection process, which can affect the living conditions of nearby residents. This qualitative evaluation underscores the importance of considering social impacts in addition to environmental and economic factors when deciding on waste management solutions for urban areas.

Further there are some practical differences between the different solutions for all the actors, residents, waste collection workers and the municipality. For the system users (residents), all solutions require users to bring waste to a designated drop-off point (waste room, chute or container). For the waste collection workers, vacuum system and underground container are less physically demanding for workers compared to indoor bins. Indoor bins are physically demanding due to frequent handling of bins. For the municipality, vacuum systems and underground containers have high flexibility for adapting to future regulations, such as adding separate collection units for new waste types.

For each stakeholder group identified, relevant indicators were assessed qualitatively. Based on the qualitative assessment, a colour code was given, where pink indicates large negative impacts, yellow indicates some negative impacts, whereas grey indicates low negative impacts (Table 3).

**Table 3.** Qualitative assessment of the three collection alternatives for selected indicators for identified stakeholder groups

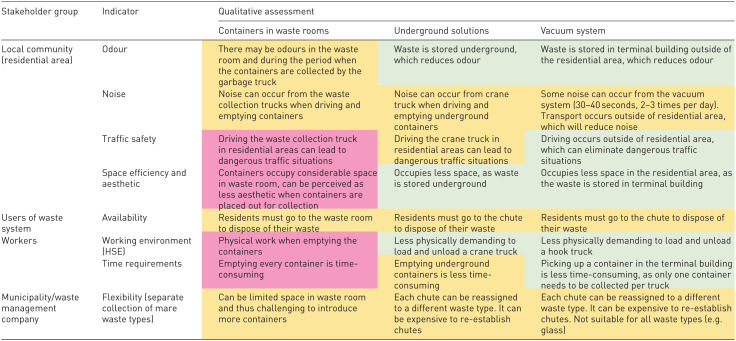

Pink has large negative impact, yellow some negative effect and grey has low negative effect.

### Area analysis: Bjørvika

In evaluating waste collection solutions for the Bjørvika area, two distinct scenarios were analysed, each encompassing the management of waste for approximately 2500 homes.

Scenario 1 envisions the implementation of a comprehensive vacuum system covering all 2500 residences. This approach centralizes waste collection through an integrated network of underground pipelines and chutes, efficiently transporting waste to a centralized terminal for sorting and processing. The vacuum system is designed to minimize the environmental footprint, particularly in terms of odour and noise, given that the bulk of waste handling and transport occurs outside residential zones.

**Figure 3.**
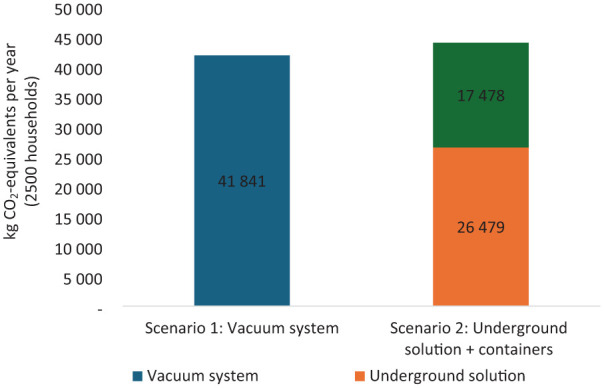
Potential climate impact for the two scenarios kilogram of CO_2_-equivalents per year (2500 households).

**Figure 4.**
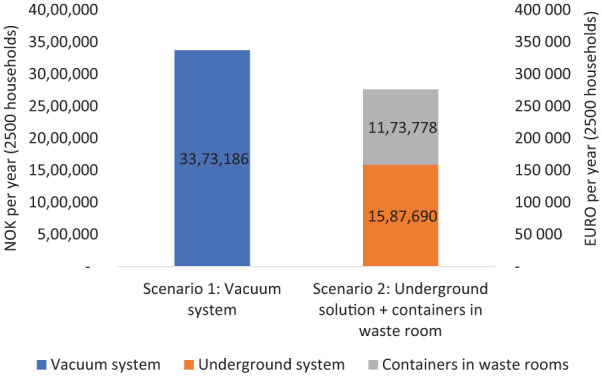
Total costs for the two scenarios in NOK and Euro per year (2500 households). NOK: Norwegian kroner.

Scenario 2 proposes a hybrid solution that combines different methods tailored to specific sub-regions within the Bjørvika area. This scenario allocates underground containers to service 1500 homes located in Grønlikaia. These containers store waste underground, thus reducing odour and visual clutter above ground. Meanwhile, the remaining 1000 homes in Bispevika are equipped with indoor bins, positioned within shared waste rooms for convenience. This dual-method approach seeks to optimize the strengths of each collection system according to the geographical and infrastructural context of the sub-areas involved, aiming to balance economic, environmental and social considerations effectively across the entire community. Each scenario represents a targeted strategy to achieve efficient and sustainable waste management tailored to the unique requirements of the Bjørvika area’s urban landscape (Figures 3 and 4).

#### Emissions

The vacuum system achieves a slightly lower climate impact due to more efficient collection and transportation.

#### Costs

The vacuum system is 22% more expensive, increased connections (e.g. commercial activities) could reduce costs.

## Results for the three collection solutions

Figure 1 illustrates the potential climate impact of the three collection solutions, vacuum systems, underground containers and indoor bins measured in kilograms of CO₂-equivalents per household per year.

The analysis reveals that while vacuum systems provide the lowest climate impact among the waste collection solutions assessed, the overall differences in environmental impact among the options remain relatively minor under the assumptions applied in the study. Each waste collection system has distinct factors contributing to its emissions profile.

For vacuum systems, the principal sources of emissions are related to the initial investment in infrastructure, specifically the construction of the pipe network and the terminal building. These emissions are amortized over the lifespan of the system, reflecting a significant upfront environmental burden.

In the case of underground containers, the emissions are predominantly influenced by the production of the collection units. The manufacturing process of these units poses the largest environmental burden. Although the infrastructure-related excavation works necessary for installing these containers is accounted for, it exerts only a marginal effect on the overall emissions results.

Regarding indoor bins, the emissions profile is mainly driven by transportation activities associated with collection operations. This is followed closely by emissions from the system’s operation, which include the use of electric forklifts for manoeuvring the bins during waste collection processes.

These insights underscore the need to consider specific emissions sources and lifecycle phases when evaluating the environmental impacts of various waste management systems, highlighting areas where efficiency improvements could be targeted.

Figure 2 illustrates the lifecycle costs of the three collection solutions in NOK per household per year.

In examining the cost dynamics of different waste collection systems, the study identifies distinct cost drivers for each method. For vacuum systems, infrastructure constitutes the primary expense, encompassing the establishment of the terminal buildings and the extensive network of pipes required for operation. This significant upfront investment represents the largest cost component for this system. In contrast, for underground containers, the production and installation of the collection units themselves account for the majority of costs, reflecting their material and logistical requirements. Meanwhile, indoor bins incur their highest costs from transportation operations, involving collection trucks, along with the ongoing maintenance and management of the bins themselves.

For system users, namely the residents, all the solutions necessitate that they transport their waste to designated drop-off points, such as waste rooms, chutes or containers, requiring a degree of engagement and mobility.

From the perspective of waste collection workers, vacuum systems and underground containers reduce the physical demands typically associated with waste collection, making them less strenuous compared to the more labour-intensive handling required for indoor bins.

Municipalities and waste management companies benefit from the adaptability of vacuum systems and underground containers, which offer significant flexibility for integrating new regulations, such as the addition of separate collection units for newly categorized waste types, thereby future-proofing waste management strategies.

Two scenarios for the Bjørvika area were thoroughly analysed to assess the practical implementation of these waste collection systems. Scenario 1 envisages the deployment of a vacuum system throughout all 2500 homes, leveraging its comprehensive infrastructure for centralized waste management. On the other hand, scenario 2 proposes a combination of methods: employing underground containers for the 1500 homes in Grønlikaia and utilizing indoor bins for the remaining 1000 homes within Bispevika. Each scenario presents its own set of advantages and challenges, seeking to optimize waste management practices in a manner tailored to the unique urban context of the Bjørvika area.

- Climate impact: Vacuum system has 5% lower CO₂-equivalents compared with scenario 2.

The vacuum system achieves a slightly lower climate impact due to more efficient collection and transportation.

- Costs: Vacuum system has 22% higher cost compared with scenario 2.

While the vacuum system is more expensive, increased connections (e.g. commercial activities) could reduce costs.

Recent advances in MSW research underscore the necessity of critical comparison and scenario-based modelling to ensure robust and policy-relevant conclusions. Bala et al. (2021) introduced the FENIX predictive LCA model, which enables detailed scenario analysis of collection system parameters such as container number, collection frequency and route characteristics. Their findings demonstrate that fixed average fuel consumption factors, commonly used in legacy models, can lead to significant inaccuracies when predicting environmental impacts, especially under varying operational conditions. By systematically varying key parameters, Bala et al. (2021) revealed that changes in collection frequency or working day duration can alter fuel consumption and emissions by over 100%, highlighting the importance of scenario modelling for both scalability and transferability to commercial waste systems. This approach directly addresses the limitations identified in the present study, where deterministic assumptions may obscure the potential range of real-world outcomes and hinder the exploration of integration with larger, more complex waste management infrastructures.

Moreover, the taxonomy developed by Rodrigues et al. (2016) provides a standardized framework for describing and benchmarking waste collection systems, facilitating meaningful comparison across diverse urban contexts. Their work, alongside the robust multi-objective modelling of Heidari et al. (2019), demonstrates the value of integrating economic, environmental and social criteria, as well as explicit sensitivity and uncertainty analyses, into MSW system design. Heidari et al. (2019) showed that improvements in waste separation efficiency can yield double-digit gains in sustainability indicators, and that scenario analysis is essential for evaluating system resilience under uncertainty. Complementing these approaches, Ghose et al. (2006) illustrated the operational benefits of Geographically information System (GIS)-based routing and bin placement optimization, which can be incorporated into scenario modelling to assess commercial scalability. Collectively, these studies suggest that future iterations of this work should incorporate scenario-based comparative analysis, leveraging standardized taxonomies and robust optimization frameworks to enhance the generalizability and practical relevance of the findings.

Although the present study provides a comprehensive overview of collection and transportation strategies for MSW, it is important to acknowledge a key methodological limitation: the absence of a formal sensitivity or uncertainty analysis. Given the inherent variability in waste generation rates, operational costs and environmental impacts, sensitivity analysis is essential for assessing the robustness of model outcomes and policy recommendations. Recent advances in MSW modelling, such as those by Jin et al. (2019) and Yadav et al. (2018), have demonstrated the value of incorporating interval analysis and fuzzy logic to explicitly address uncertainty in facility location and flow allocation models.

These approaches allow for the systematic evaluation of how changes in input parameters, such as waste quantities, transportation distances or cost coefficients, can influence optimal solutions and system performance. By contrast, the current study relies on deterministic assumptions, which, while providing clarity, may not fully capture the range of plausible real-world scenarios. Future research should therefore prioritize the integration of sensitivity and uncertainty analyses, employing techniques such as scenario analysis, Monte Carlo simulation or robust optimization, to enhance the credibility and applicability of model-based recommendations. Critically reflecting on this shortcoming not only aligns with the evolving standards in the field but also underscores the need for transparent and adaptive decision-support tools in sustainable MSW management (Jin et al., 2019; Yadav et al., 2018).

## Conclusion and further work

In conclusion, the comprehensive analysis of waste collection solutions, encompassing vacuum systems, underground containers and indoor bins, alongside the specific scenarios evaluated for the Bjørvika area, underscores the complexity of determining the optimal waste management approach. Several key findings have emerged from this study.

From an environmental perspective, the differences in greenhouse gas emissions across the three collection solutions are relatively minor. In vacuum systems, the establishment of the pipe network and terminal building represents the most significant source of emissions. Conversely, the emissions from underground containers are largely contingent upon the number of units, with production being the primary contributing factor. For indoor bins, emissions are predominantly driven by transport and the operation of the system, including the use of forklifts.

Economically, both vacuum systems and indoor bins with three separate collection units are more costly compared to other options within the parameters of this analysis. Notably, within the context of the Bjørvika analysis, although vacuum systems demonstrated a 5% reduction in climate impact for 2500 homes, they incurred 22% higher costs compared to the hybrid solution of underground containers and indoor bins.

Scalability and flexibility further inform these assessments. The viability of vacuum systems is particularly sensitive to the number of households they serve, with the inclusion of commercial waste also providing potential cost efficiencies. Increasing the connected homes from 2500 to 3100 households results in a 14% reduction in costs, positioning vacuum systems more competitively against underground containers. Additionally, developing fair cost distribution models for shared use of terminal buildings offers another avenue for optimization.

Social implications of these systems indicate that vacuum systems provide specific advantages such as reduced odour and noise, alongside enhanced traffic safety due to externalized waste transport. They also pose fewer physical demands on collection personnel and caretakers relative to indoor bins. However, given the study’s simplified approach to social impacts, further detailed assessment using recognized methodologies, such as the S-LCA, is recommended for comprehensive communication of findings.

Lastly, space and land use considerations emerge as critical, particularly in urban contexts where spatial constraints can significantly influence the living environment. Although underground containers necessitate ample space for crane access, indoor bins utilize valuable space that could serve alternative, beneficial purposes like bike parking or storage.

Several recommendations are proposed for future research. A deeper analysis into the scalability of vacuum systems, focusing on their integration with additional residential units and commercial entities, could yield important insights. Developing cost-sharing models for multifunctional terminal buildings could ensure equitable expense allocation. Future studies should incorporate land use impact indicators to better assess the influence of waste collection solutions on urban environments. Additionally, a comprehensive evaluation of social aspects through established methodologies such as S-LCA would enhance understanding of stakeholder impacts, thus providing municipalities and stakeholders with a more nuanced and informed basis for decision-making. Through these endeavours, a more robust understanding of the sustainability of various waste collection solutions will be developed, facilitating informed choices in urban waste management.

Two distinct analyses underpin this study. The first involves a comparative assessment of three collection solutions: indoor bins, underground containers and vacuum systems, with results expressed on a per household per annum basis. The second analysis examines two potential area scenarios within Bjørvika: scenario 1 envisions vacuum systems serving all 2500 homes, whereas scenario 2 proposes a hybrid approach of underground containers for 1500 homes and indoor bins for 1000 homes, evaluating performance for the total area annually.

The sustainability assessment is comprehensive, integrating three key dimensions: climate impact, cost analysis and a simplified qualitative evaluation of social aspects. The climate impact is measured via LCA, whereas the cost analysis extends across the entire lifecycle of the collection systems. Social considerations, though simplified, are derived from literature reviews and collaboration with key stakeholders.

A holistic comparison of waste management technologies requires not only the separate reporting of CO_2_ emissions and costs but also their integration through a transparent weighting or multi-criteria decision analysis. As demonstrated by Heidari et al. (2019), robust multi-objective models that explicitly weigh environmental and economic objectives enable decision-makers to identify solutions that best balance sustainability trade-offs. Similarly, Bala et al. (2021) highlighted that scenario-based LCA models can be extended to compare alternative collection strategies using composite indicators, whereas Rodrigues et al. (2016) emphasized the importance of standardized system descriptions for meaningful benchmarking.

To advance the field and support more actionable recommendations, future studies should employ integrated assessment frameworks, such as multi-criteria decision analysis or weighted scoring, that explicitly combine CO_2_ emissions and cost. This approach would facilitate a holistic evaluation of the three technologies, ensuring that trade-offs are transparent and aligned with policy or stakeholder priorities. Such a comprehensive comparison represents a critical next step for research and practice in sustainable waste management.

Findings reveal that the most suitable waste collection solution cannot be universally applied; rather, it depends heavily on site-specific conditions that significantly influence outcomes. Key observations include that vacuum systems, although environmentally advantageous with the lowest climate impact, exhibit small differences relative to other systems. The establishment of the pipe system and terminal facilities are major emission sources for vacuum systems, whereas for underground solutions, emissions are largely affected by the number of collection units. Indoor bins exhibit the highest emissions from transport and operational activities. In terms of cost, vacuum systems emerge as one of the pricier options. However, sensitivity analyses suggest these costs could align more closely with those of underground solutions if the number of connected homes increases, showcasing potential economies of scale inherent to vacuum systems. Socially, vacuum systems offer benefits such as reduced odour, noise and improved traffic safety when terminals are placed outside residential areas.

## Supplemental Material

sj-docx-1-wmr-10.1177_0734242X251385787 – Supplemental material for Sustainability assessment of waste vacuum systems as a collection solution Supplemental material, sj-docx-1-wmr-10.1177_0734242X251385787 for Sustainability assessment of waste vacuum systems as a collection solution by Eirill Bø and Kari-Anne Lyng in Waste Management & Research
