# Supplementary material for: Sustainability assessment of waste vacuum systems as a collection solution
Source: Waste Manag Res. 2025 Nov 13;44(4):465–75. doi: 10.1177/0734242X251385787 (PMC12987990; doi:10.1177/0734242X251385787)
Supplement: sj-docx-1-wmr-10.1177_0734242X251385787 – Supplemental material for Sustainability assessment of waste vacuum systems as a collection solution [file sj-docx-1-wmr-10.1177_0734242X251385787.docx]

**Supplementary material:**

Waste Vacuum Collection System - Input Factors (Serving 2,500 Households)

# Waste Inlets for Vacuum System

| Input Factor | Value | Unit | Source/Explanation |
| --- | --- | --- | --- |
| Number of inlets | 62 | units | Provided by Envac. Designed as the minimum required. |
| Lifetime | 20 | years | Provided by Envac. |
| Materials | 0,572 | tonnes/inlet | Steel, low alloyed, hot rolled, processed. Provided by Envac. |
| Installation cost per inlet | 163.000 | NOK/inlet | Provided by Envac, based on component pricing. |

# Operation of the Vacuum Waste System

| Input Factor | Value | Unit | Source/Explanation |
| --- | --- | --- | --- |
| Energy use (vacuum system) | 299.000 | kWh/year | Envac states 100 kWh/ton of waste. Waste quantity is 2,990 tonnes. Based on Norwegian electricity mix. |
| Heating and fan energy (terminal) | 60.893 | kWh/year | Estimated energy use for an 825 m² terminal building. Provided by Envac. |
| Electricity price | 1,5 | NOK/kWh | Same base assumption as Olsen and Kyllingstad (2023). |

# Transport of Waste from Vacuum Terminal

| Input Factor | Value | Unit | Source/Explanation |
| --- | --- | --- | --- |
| Fuel consumption (hook-lift truck) | 0,37 | kg biogas/km | Provided by Oslo REG |
| Truck lifetime | 10 | years | Provided by Oslo REG, with some uncertainty |
| Container capacity | 10 | tonnes | Provided by Oslo REG |
| Round-trip distance | 8,5 x 2 | km | Distance between Grønlikaia and Haraldrud |
| Number of trips | 301 | trips/year | Calculated by dividing waste amount by payload |

# Number of Annual Truck Trips per Waste Type

| Waste Type | Tonnes /Year | Number of Containers = Trips |
| --- | --- | --- |
| Residual Waste | 1144 | 115 |
| Plastic | 390 | 39 |
| Food Waste | 384 | 39 |
| Cardboard and Paper | 1073 | 108 |
| Total | 2990 | 301 |

# Infrastructure Inputs for Vacuum Collection System

| Input Factor | Value | Unit | Source/Explanation |
| --- | --- | --- | --- |
| Investment cost (terminal & system) | 50,1 million | NOK | Provided by Envac; includes terminal, vacuum system, and related infrastructure. |
| Terminal lifetime | 60 | years | Provided by Envac |
| Terminal size | 840 | m² | Generic data for environmental load of a production hall (Building hall, market for, GLO). |
| Number of containers in terminal | 4 | units | Provided by Envac |
| Investment per container | 562.500 | NOK | 450,000 excl. VAT. Provided by Envac. |
| Material per container | 4.4 | tonnes | Steel, low alloyed, hot rolled, processed. Provided by Envac. |
| Container lifetime | 20 | years | Provided by Envac |
| Pipe system | 890 | meters | Steel, 508 mm outer diameter. Provided by Envac. |
| Pipe system lifetime | 60 | years | Provided by Envac |
